# Identification of the core genes in Randall’s plaque of kidney stone and immune infiltration with WGCNA network

**DOI:** 10.3389/fgene.2023.1048919

**Published:** 2023-02-01

**Authors:** Lingyun Yu, Gefei Li, Shiyao Jin, Jiahong Su, Shoulin Li

**Affiliations:** ^1^ Department of Urology, Shenzhen Children’s Hospital, Shenzhen, Guangdong, China; ^2^ Department of Cardiovascular Surgery, Shenzhen, Guangdong, China

**Keywords:** WGCNA (weighted gene co-expression network analyses), immune infiltration, kidney stone, renal calculi, CIBERSORT, Randall’s plaque

## Abstract

**Background:** Randall’s plaque is regarded as the precursor lesion of lithiasis. However, traditional bioinformatic analysis is limited and ignores the relationship with immune response. To investigate the underlying calculi formation mechanism, we introduced innovative algorithms to expand our understanding of kidney stone disease.

**Methods:** We downloaded the GSE73680 series matrix from the Gene Expression Omnibus (GEO) related to CaOx formation and excluded one patient, GSE116860. In the RStudio (R version 4.1.1) platform, the differentially expressed genes (DEGs) were identified with the limma package for GO/KEGG/GSEA analysis in the clusterProfiler package. Furthermore, high-correlated gene co-expression modules were confirmed by the WGCNA package to establish a protein–protein interaction (PPI) network. Finally, the CaOx samples were processed by the CIBERSORT algorithm to anchor the key immune cells group and verified in the validation series matrix GSE117518.

**Results:** The study identified 840 upregulated and 1065 downregulated genes. The GO/KEGG results revealed fiber-related or adhesion-related terms and several pathways in addition to various diseases identified from the DO analysis. Moreover, WGCNA selected highly correlated modules to construct a PPI network. Finally, 16 types of immune cells are thought to participate in urolithiasis pathology and are related to hub genes in the PPI network that are proven significant in the validation series matrix GSE117518.

**Conclusion:** Randall’s plaque may relate to genes *DCN*, *LUM*, and *P4HA2* and M2 macrophages and resting mast immune cells. These findings could serve as potential biomarkers and provide new research directions.

## 1 Introduction

Kidney stone (KS), as a specific disease in adult and pediatric urology, is mainly caused by abnormal renal deposition of crystals (CaOx, CaP, and uric acid) in the calyx, pelvis, and ureteropelvic junction (UPJ). The prevalence of KS varies globally, ranging from 7%–13% in North America, 5%–9% in Europe, and 1%–5% in Asia (Sorokin et al., 2017). Randall’s plaque, first described by Randall in 1937 (Randall, 1937), is considered the precalculus lesion to renal calculi, and most renal lithiases are calcium oxalate (CaOx). The precalculus lesions were classified into Randall’s plaque and Randall’s plugs, termed Randall’s plaque type I and II. The former originates from the calcium phosphate (CaP) crystal cores deposited in interstitium at the basement membrane of the Henle loop through the fixed-particle mechanism, and the latter derives from calcium phosphate plugs blocked in Bellini’s ducts by a free-particle mechanism (Kok and Khan, 1994; Daudon et al., 2015; Khan et al., 2016). Several molecular mechanisms involving osteogenic calcification (Gay et al., 2020) or reactive oxygen species (ROS) (Liu and Yu, 2019) are proposed to explain the transformation from Randall’s plaque to kidney stone, which generally comprises nucleation, growth, and aggregation (Khan, 2018). However, the critical pathogenic genes still have not yet been fully explored.

In addition, recent studies have drawn more public attention to immune reactions and inflammation in nephrolithiasis (Khan et al., 2021). Until now, M1/M2 macrophage polarization (Taguchi et al., 2016) has been researched as a regulatory segment to engulf crystals, while less attention has been given to other immune cells, like the γδT cell (Zhu et al., 2019). Moreover, the correlation between gene expression and active immune cells remains unknown.

To explore core genes and immune cells in Randall’s plaque progression, weighted gene co-expression network analysis (WGCNA) (Langfelder and Horvath, 2008), instead of traditional microarray analysis, is applied to identify gene modules based on gene expression levels highly correlated with samples. Combined with CIBERSORT (Newman et al., 2015), an immune cell algorithm that could estimate immune cell abundance in samples, potential immune cells, and regulatory networks will be revealed for subsequent analysis in correlation with key genes from the gene module.

In this study, the expression matrices of 24 CaOx Randall’s plaque samples and six healthy samples are downloaded from GSE73680 in the public GEO database for analysis. Then, the Kyoto Encyclopedia of Genes and Genomes (KEGG) pathways and gene ontology (GO) and disease ontology (DO) terms are utilized to describe the overall gene expression status. The core genes from the gene module and the proportions of immune cells are respectively computed by WGCNA and CIBERSORT, and correlation among calculated results is assessed. Finally, we used samples from GSE117518 to validate the expression of the core genes.

## 2 Materials and methods

### 2.1 Microarray data

**FIGURE 1:**
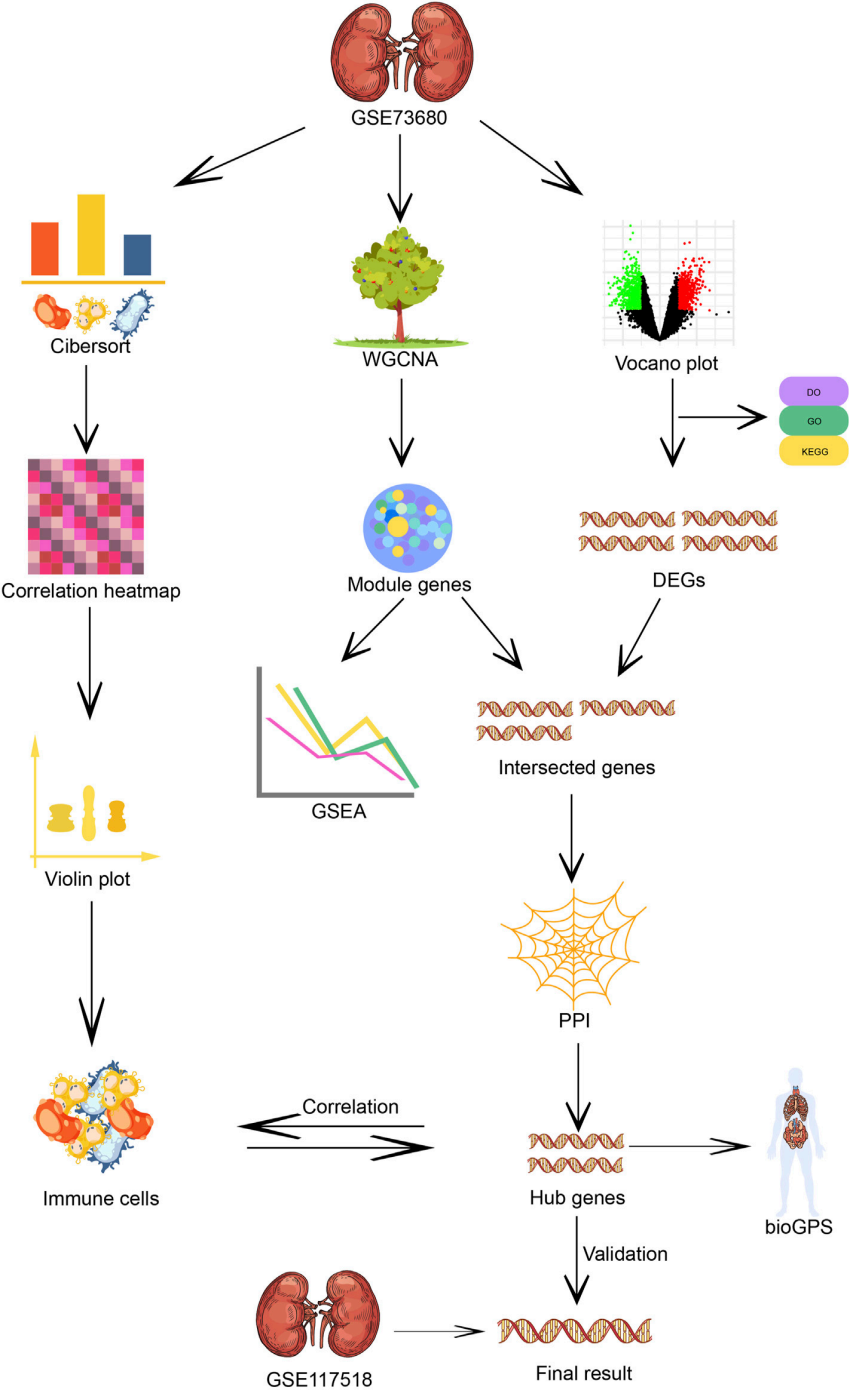
Flow chart of immune infiltration, WG-CNA algorithm and core genes screening of the microarray analysis process.

We downloaded the public GEO normalized series matrices GSE73680 and GSE117518 from the NCBI database. Three relative data sets series are anchored by retrieval strategy “(CaOx)” AND “*Homo sapiens*” [porgn:__txid9606].” We excluded GSE116860 due to its unique origin. GSE73680 contained six normal samples and 13 CaOx plaque mucosa samples. Eleven CaOx>CaP plaque samples were also attributed to the CaOx-RP group for predominant CaOx composition according to the information described in the microarray provider’s article (Taguchi et al., 2017). GSE117518 was composed of three control and three CaOx-RP samples (Figure 1).

### 2.2 Filtering differentially expressed genes (DEGs) and gene ontology (GO)/Kyoto Encyclopedia of Genes and Genomes (KEGG)/disease ontology (DO)/gene set enrichment analysis (GSEA) analysis

The GSE73680 and GSE117518 expression matrices were log2 transformed and normalized by the R function “normalizeBetweenArrays.” Furthermore, the GPL17077 annotation table was downloaded to convert gene probes to symbols in GSE73680, whereas the validation data set GSE117518 gene conversion was completed by the R package “idmap3” due to lack of a probe-ID corresponding relationship in GPL21827. On the basis that the *p*-value < 0.05 and the log2 fold change (logFC) cut-off absolute value > 1, GSE73680 successfully adopted the LIMMA package to select DEGs. Then, the DEG volcano picture produced by R packages “ggplot2” and “ggrepel” was imported to GO/KEGG/DO for ranking gene functions and pathways by applying the “clusterProfiler” (Yu et al., 2012), “GOplot” (Walter et al., 2015), and “DOSE” R packages.

### 2.3 Weighted gene co-expression network analysis (WGCNA) construction and identification of trait-related modules and hub genes in protein–protein interaction (PPI) network

In the first step, all genes with 25% variance or more were selected, and WGCNA analysis was performed. After removing outlier samples in the cluster tree using the “flashclust” R package, we reserved residual samples for subsequent processing. To balance the mean connectivity and relationship degree square (named “R^2” and generally higher than 0.8), a rational soft power threshold was recommended for the adjacency matrix through the “pickSoftThreshold” function built into the “WGCNA” package (Langfelder and Horvath, 2008) from 1 to 20.

The second step transformed the adjacency matrix into a topological overlap matrix (TOM) and TOM-based dissimilarity (1-TOM). The “hclust” (hierarchical clustering) R function calculated the average linkage hierarchical clustering by a TOM-based dissimilarity measure with a minimum gene volume of 50 for the gene dendrogram. Genes with similar expression profiles could be attributed to various gene modules by employing the “cutreeDynamic” (dynamic tree cut) function.

The third step sets the module’s correlation coefficient threshold and merges homologous modules in a dynamic cluster tree. The following procedure evaluates the expression–trait correlation plotted in various random color modules. The highest correlated module eigengene (ME) was identified as the key gene group with gene significance (GS) *p* < 0.05. A scatter diagram with corresponding colored modules identifying the gene points was created, and GSEA analysis was executed using the “enrichplot” package.

### 2.4 PPI network construction and identification of hub genes

In the online platform STRING (version 11.0; www.string-db.org) (Szklarczyk et al., 2021), ME and DEG intersecting genes identified by the “nVennR” package were imported to visualize the protein–protein interaction (PPI) with a minimum interaction score ≥ 0.9. To reveal hub gene locations in PPI network, STRING interaction results were evaluated by Cytoscape software (version 3.9.0) to screen out gene clusters. We sorted relative hub genes with the “Degree” method in the plug-in unit cytoHubba. The top 10 hub genes were located in high-expression sites using BioGPS (biogps.org/#goto=welcome) (Wu et al., 2016) and visualized in Rawgraphs (version 2.0 beta; rawgraphs.io/).

### 2.5 CIBERSORT algorithm analysis of immune cell infiltration in and correlation among immune cells

To explore the mechanism of immune cells in the formation of Randall’s plaque, we adopted the CIBERSORT algorithm (Newman et al., 2015) to calculate the percentage of 22 background immune cells in samples. These background immune cells were as follows: memory B cells, naive B cells, naive CD4^+^ T cells, CD8^+^ T cells, activated memory CD4^+^ T cells, resting memory CD4^+^ T cells, Tfh, regulatory T cells, gamma–delta T cells, plasma cells, resting natural killer (NK) cells, activated NK cells, monocytes, M0 macrophages, M1 macrophages, M2 macrophages, resting mast cells, activated mast cells, resting dendritic cells, activated dendritic cells, eosinophils, and neutrophils. We extracted the CIBERSORT outcomes with *p*-value < 0.05 to estimate the correlation coefficient among positive immune cells in the “corrplot” package, and |coefficient| > 0.7 is considered highly correlated. We also used CIBERSORT outcomes to compare immune cell content between the control and RP using the “vioplot” package with *p*-values < 0.05. Finally, principal component analysis (PCA) was introduced to distinguish the RPs and normal samples by displaying corresponding ovals.

### 2.6 The correlation between hub genes and immune cells plus expression level in the validation data set

We first assessed the relationship between hub genes and immune cell groups through a lollipop plot. Then, the GSE117518 matrix was used to extract the expression of the hub genes to create a heatmap plot in the “pheatmap” R package, and the Shapiro–Wilk test was used to confirm normal distribution in these samples, although normal distribution is commonly recognized as such in log2-processed microarray data. Subsequently, the significance of hub gene expression levels in the GSE117518 samples was verified by the *t*-test, and boxplots tagged with statistical differences were created in R. Those with statistically significant differences were tagged with an asterisk.

## 3 Results

### 3.1 Identification of DEGs and enrichment analysis

**FIGURE 2:**
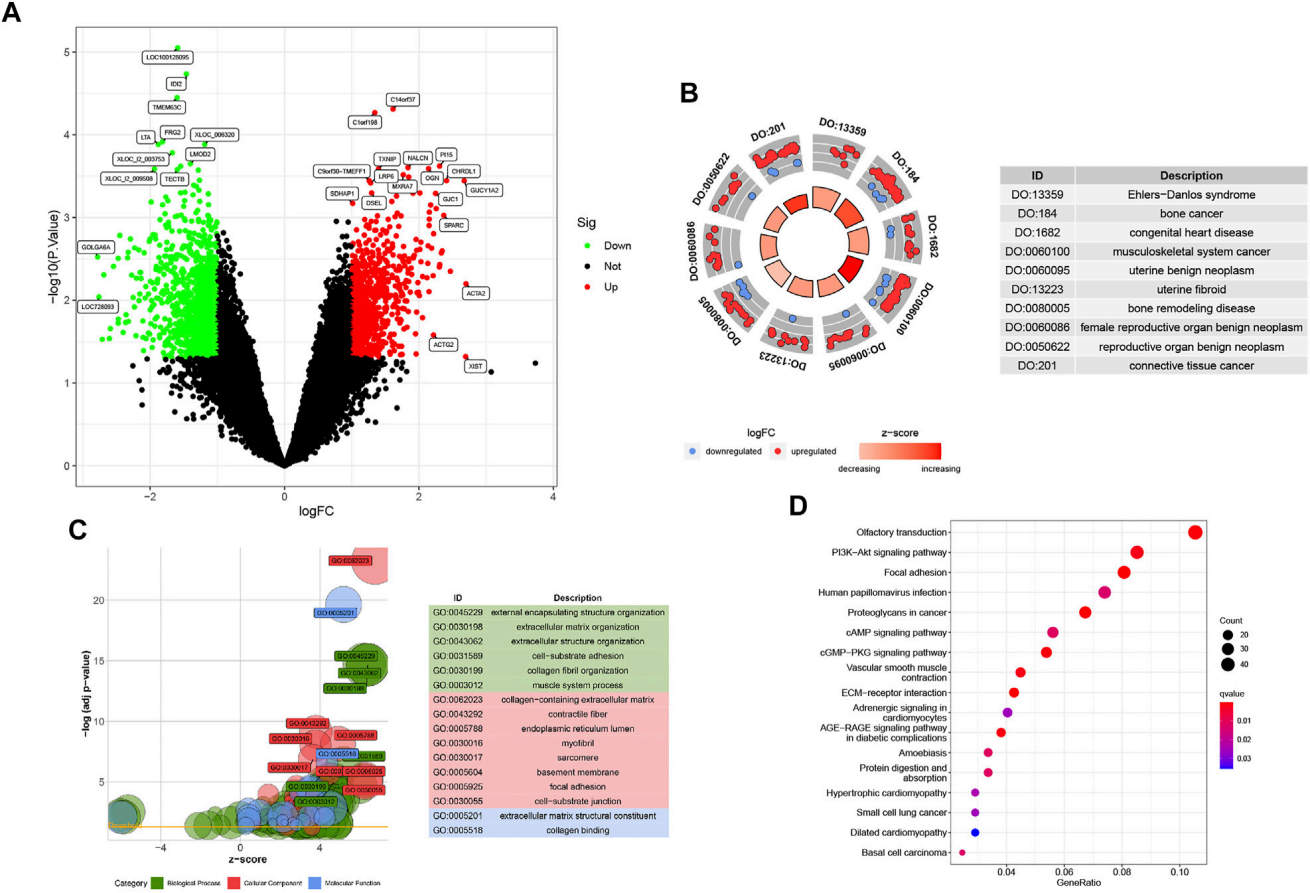
**(A)** Baseline p1 with 1905 DEGs, including 840 upregulated and 1065 downregulated genes. **(B)** DO analysis involving the top 10 related diseases. **(C)** All GO analyses for DEGs are divided into BP, CC, and MF parts. **(D)** KEGG bubble plot of Randall’s plaque pathways.

In total, 1905 DEGs are screened with gene labels in the volcano plot (Figure 2A), which contains 840 upregulated and 1065 downregulated genes in GSE73680 based on |logFC| > 1 and *p* < 0.05.

The DOcircle plot, ordered by q-value, identifies the top 10 diseases that could be related to Randall’s plaque: Ehlers−Danlos syndrome, bone cancer, congenital heart disease, musculoskeletal system cancer, uterine benign neoplasm, uterine fibroid, bone remodeling disease, female reproductive organ benign neoplasm, reproductive organ benign neoplasm, and connective tissue cancer (Figure 2B).

In GO analysis, DEGs mainly focus on external encapsulating structure organization, collagen−containing extracellular matrix, and extracellular matrix structural constituent. Other DEGs involve fiber-related or adhesion-related terms in the biological process (BP), cellular component (CC), and molecular function (MF) bubble plot (Figure 2C).

The KEGG bubble plot exhibits concentrated olfactory transduction, focal adhesion, PI3K-Akt, human papillomavirus infection, and proteoglycans in cancer pathways by the gene ratios in the DEG results (Figure 2D).

### 3.2 WGCNA analysis and module gene GSEA analysis

**FIGURE 3:**
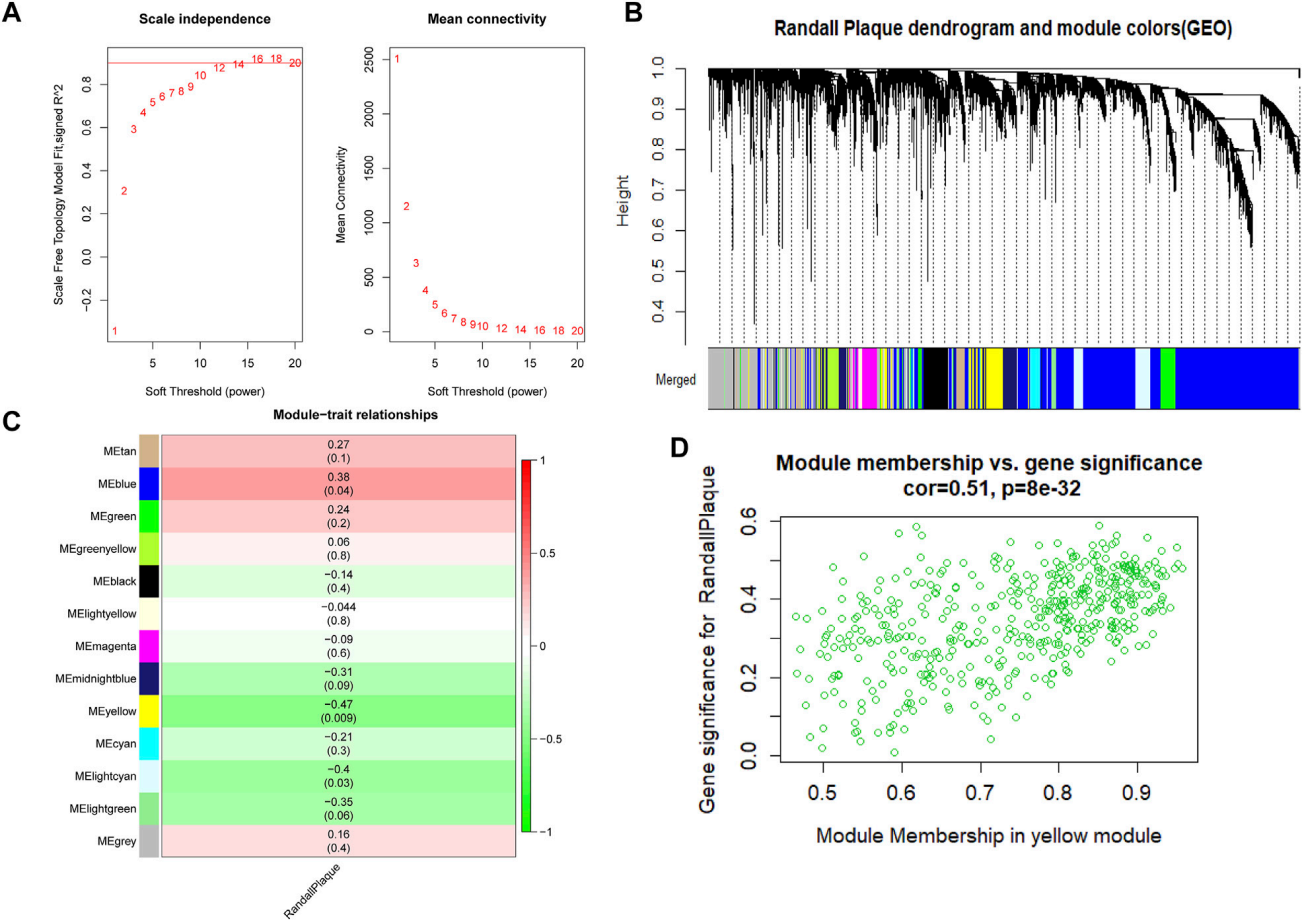
**(A)** Red line represents the reasonable soft power value in WGCNA analysis. **(B)** Randall’s plaque genes merged into various color modules in the dendrogram, and most genes are clustered in the blue module. **(C)** Various colored modules show the correlation values and *p*-values in brackets. **(D)** Scatter plot shows the degree to which genes belong to the yellow module on the *x*-axis and the coefficient of gene correlated with Randall’s plaque on the *y*-axis.

**FIGURE 4:**
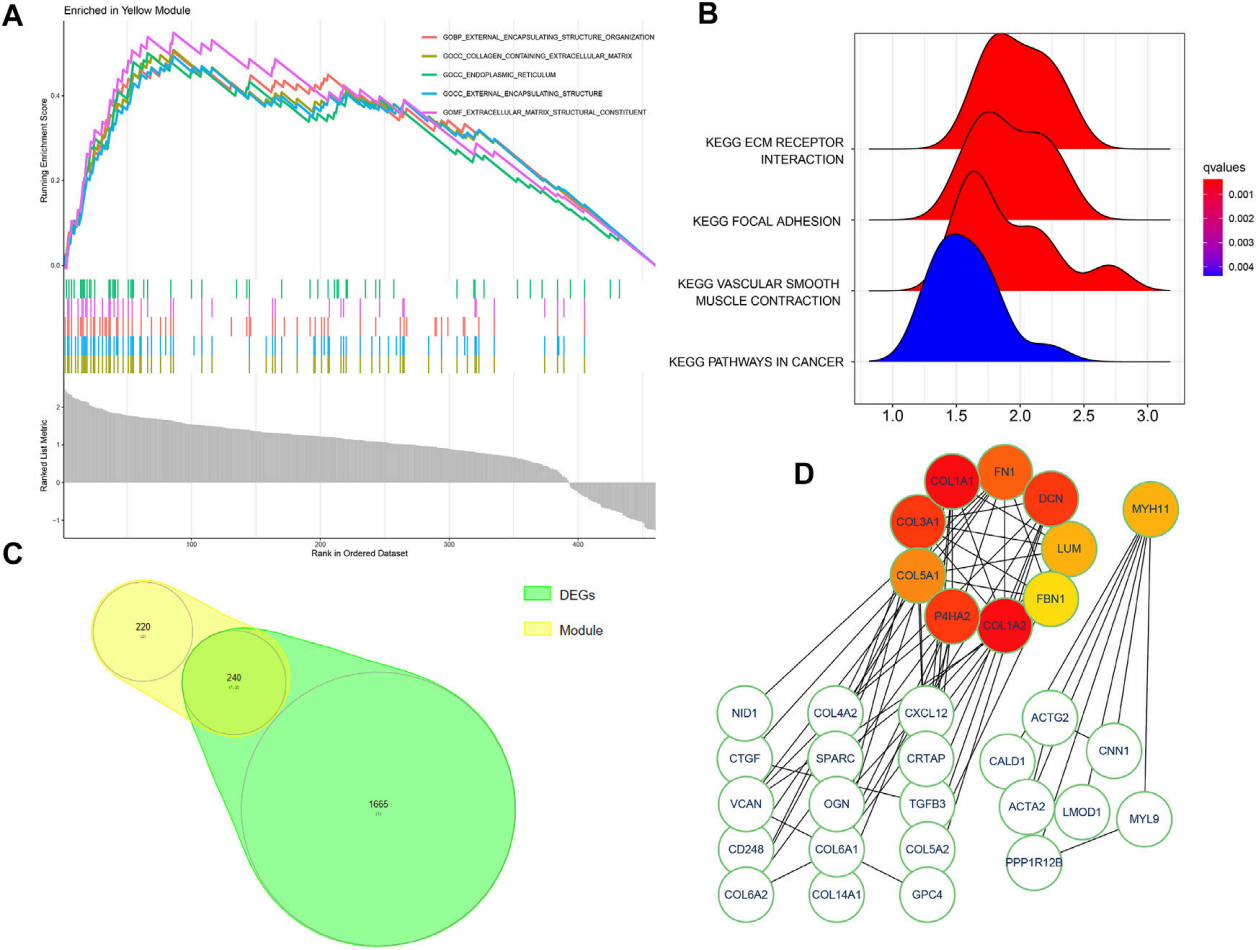
**(A,B)** GSEA analysis for the yellow module genes exported from the WGCNA method. **(C)** Intersected genes of the yellow module and DEGs. **(D)** Ten colored modules indicate hub genes and two regulatory network clusters of intersected genes.

At first, 8020 genes were reserved based on the upper 25% variance, and there was no outliner sample in the cluster analysis. The system recommended the soft power parameter of β is 12 (scale-free *R* ^2^ = 0.94) (Figure 3A) for a scale-free network. Moreover, the dynamic cut tree merged partial modules with a module correlation coefficient larger than 0.8 (Figure 3B). In the module-trait plot (Figure 3C), the yellow module shows a negative correlation coefficient with Randall’s plaque (*r* = −0.47; *p*-value = 0.09) in all 13 modules. Subsequently, the yellow module was regarded as the core module to Randall’s plaque formation, and gene-module memberships (MMs)/gene significances (GSs) were proved correlated (cor = 0.51 and P = 8e-30) in the scatter plot (Figure 3D). Additionally, GSEA analysis for the yellow module concluded that GO focuses on external encapsulating structure organization, collagen-containing extracellular matrix, endoplasmic reticulum, external encapsulating structure, and extracellular matrix structural constituent. Only four pathways are found in KEGG: ECM receptor interaction, focal adhesion, pathways in cancer, and vascular smooth muscle contraction (Figures 4A, B).

### 3.3 PPI network building and cluster genes in Cytoscape

By filtering disconnected nodes, a PPI network with 210 nodes and 97 edges was imported into the cytoHubba module to cluster 240 common genes (Figure 4C). The top 10 hub genes (*COL1A2*, *COL1A1*, *COL3A1*, *DCN*, *P4HA2*, *FN1*, *COL5A1*, *LUM*, *MYH11*, and *FBN1*) (Figure 4D) were all downregulated in Randall’s plaque and distributed in a two-network radius with a minimum connectivity degree of 7 (Supplementary Table S1). These genes are attributed to the myosin gene and collagen function genes. The collagen function genes include collagen-related genes (*COL1A2*, *COL1A1*, *COL3A1*, and *COL5A1*), decorin (DCN), fibronectin 1(FN1), lumican (LUM), and prolyl 4-hydroxylase subunit alpha 2 (P4HA2), while the myosin gene is *MYH11* (myosin heavy chain 11).

### 3.4 Immune cell distribution and differences by CIBERSORT algorithm

**FIGURE 5:**
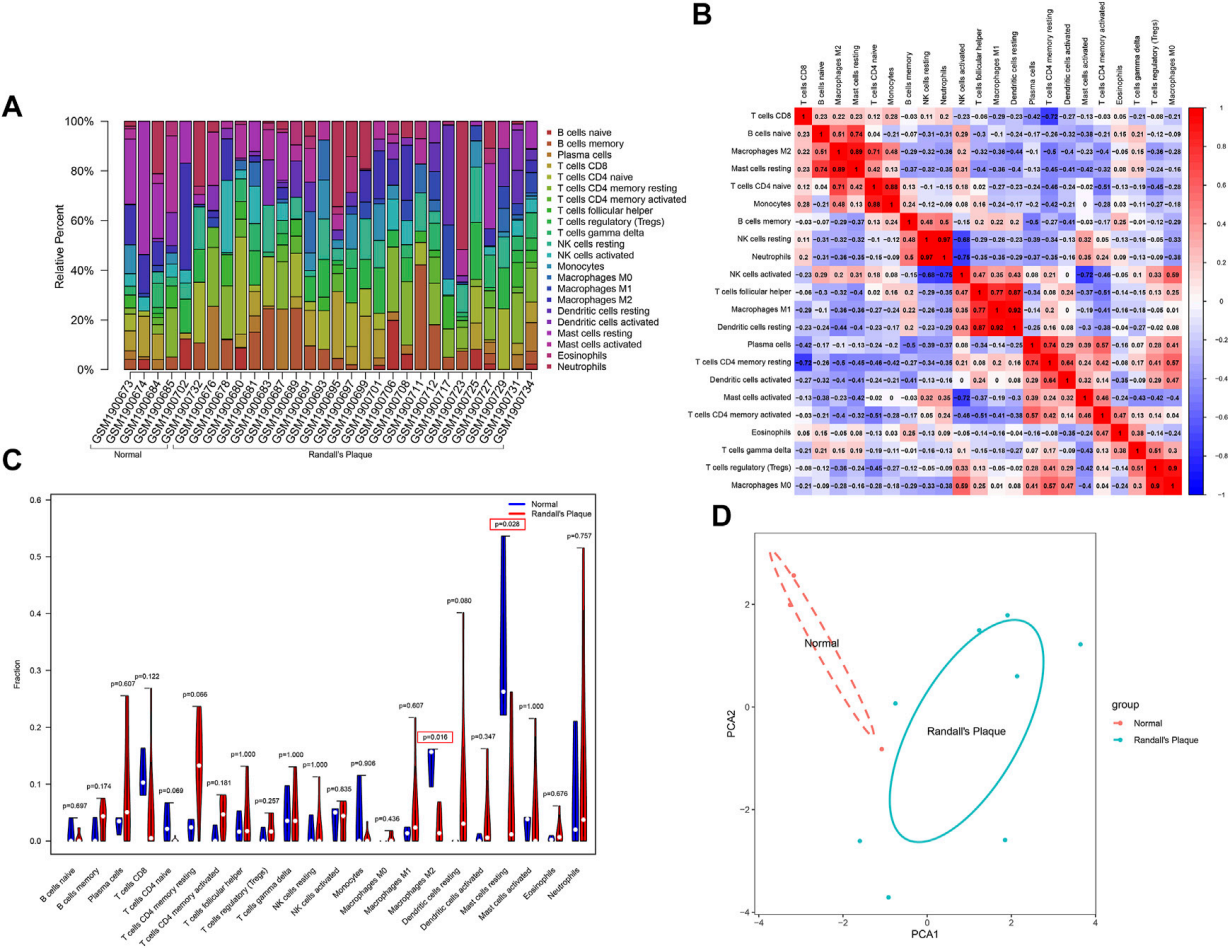
**(A)** CIBERSORT algorithm estimation of the percentages of 22 immune background cells in GSE73680. **(B)** Heatmap displays immune cell correlation among samples with *p*-values. **(C)** Violin plot shows immune cells infiltration statistical difference in normal and RP samples. **(D)** PCA analysis differentiates normal with RP samples.

The barplot (Figure 5A) shows the percentages of the 22 types of immune background cells calculated in the 30 samples. The control group exhibits a longer purple strip than the RP group. A comprehensive immune network that indicates the correlations among 11 selected samples (*p* < 0.05) was calculated by CIBERSORT11 (Figure 5B). The correlations are: neutrophils–NK cells resting (*r* = 0.97), dendritic cells resting–macrophages M1 (*r* = 0.92), macrophages M0–T cells regulatory (T regs) (*r* = 0.9), mast cells resting–macrophages M2 (*r* = 0.89), monocytes-T cells CD4 naïve (*r* = 0.88), dendritic cells resting–T cells follicular helper (*r* = 0.87), macrophages M1–T cells follicular helper (*r* = 0.77), mast cells resting–B cells naïve (*r* = 0.74), T cells CD4 memory resting–plasma cells (*r* = 0.74), T cells CD4 naïve–macrophages M2 (*r* = 0.71), neutrophils–NK cells activated (*r* = −0.75), T cells CD4 memory resting–T cells CD8 (*r* = −0.72), and mast cells activated–NK cells activated (*r* = −0.72). The violin plot (Figure 5C) shows the statistical difference (with *p* < 0.05) between the RP and control groups in M2 macrophages and resting mast cells that may interact with the aforementioned immune network to regulate Randall’s plaque progression. Finally, PCA analysis reveals the differential diagnosis among samples in the presence of specific immune cells (Figure 5D).

### 3.5 Hub gene–immune cell correlation and human tissue locations

**FIGURE 6:**
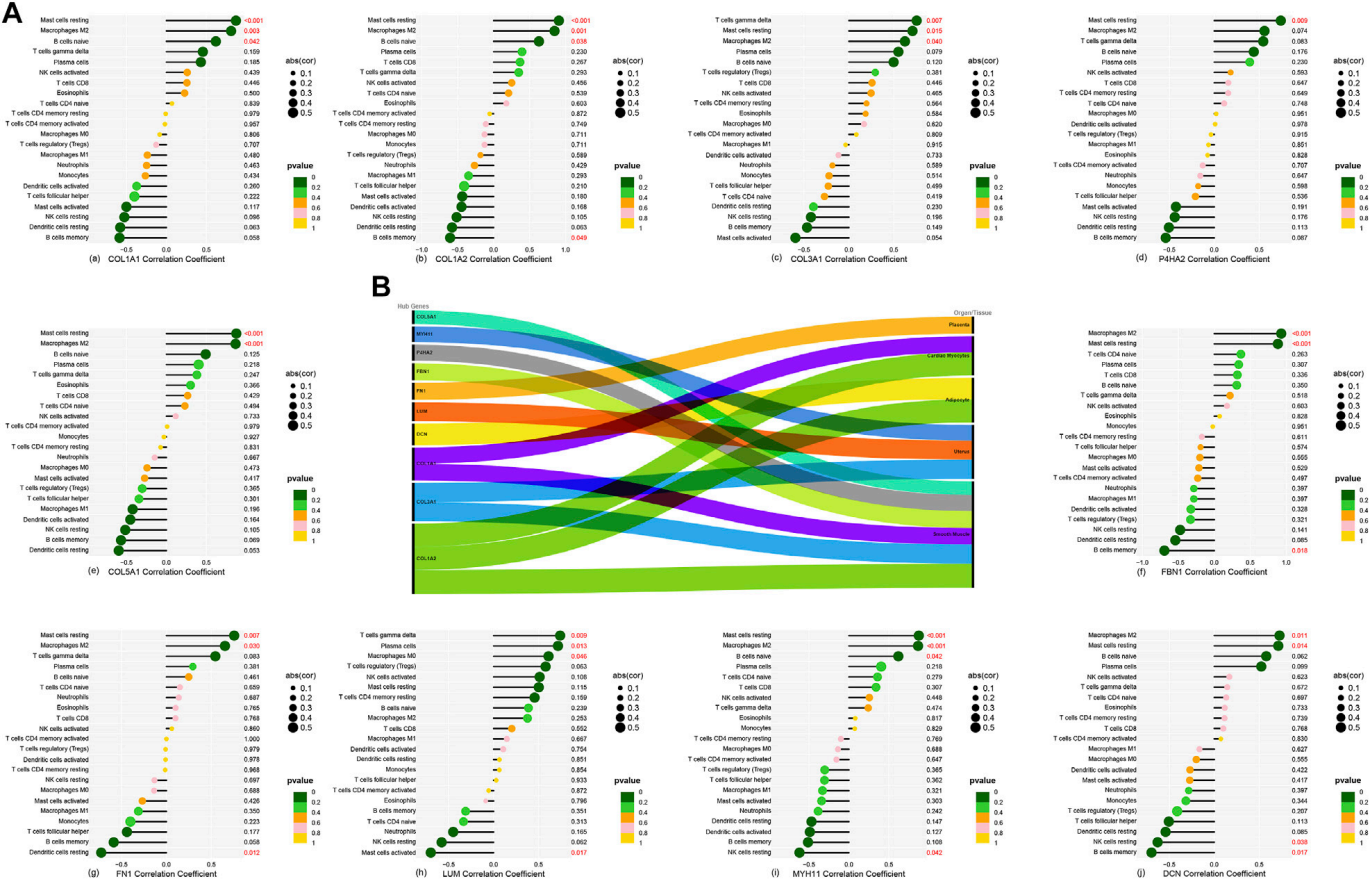
**(A-a–A-j)** Correlation between 10 hub genes and 22 background immune cells. **(B)** Ten hub genes located in the main expression sites.

A lollipop plot shows the correlations between 10 hub genes (*COL1A2*, *COL1A1*, *COL3A1*, *DCN*, *P4HA2*, *FN1*, *COL5A1*, *LUM*, *MYH11*, and *FBN1*) with 22 immune background cells (Figures 6Aa–j). All show various degrees of connection to immune cells with *p* < 0.05, which could further intensify our understanding of the relationship between immune cells and renal stones. BioGPS analysis reveals these genes are highly expressed mainly in smooth muscle and the uterus in humans (Figure 6B; Supplementary Table S2).

### 3.6 Expression level and hub gene statistical results in GSE117518

**FIGURE 7:**
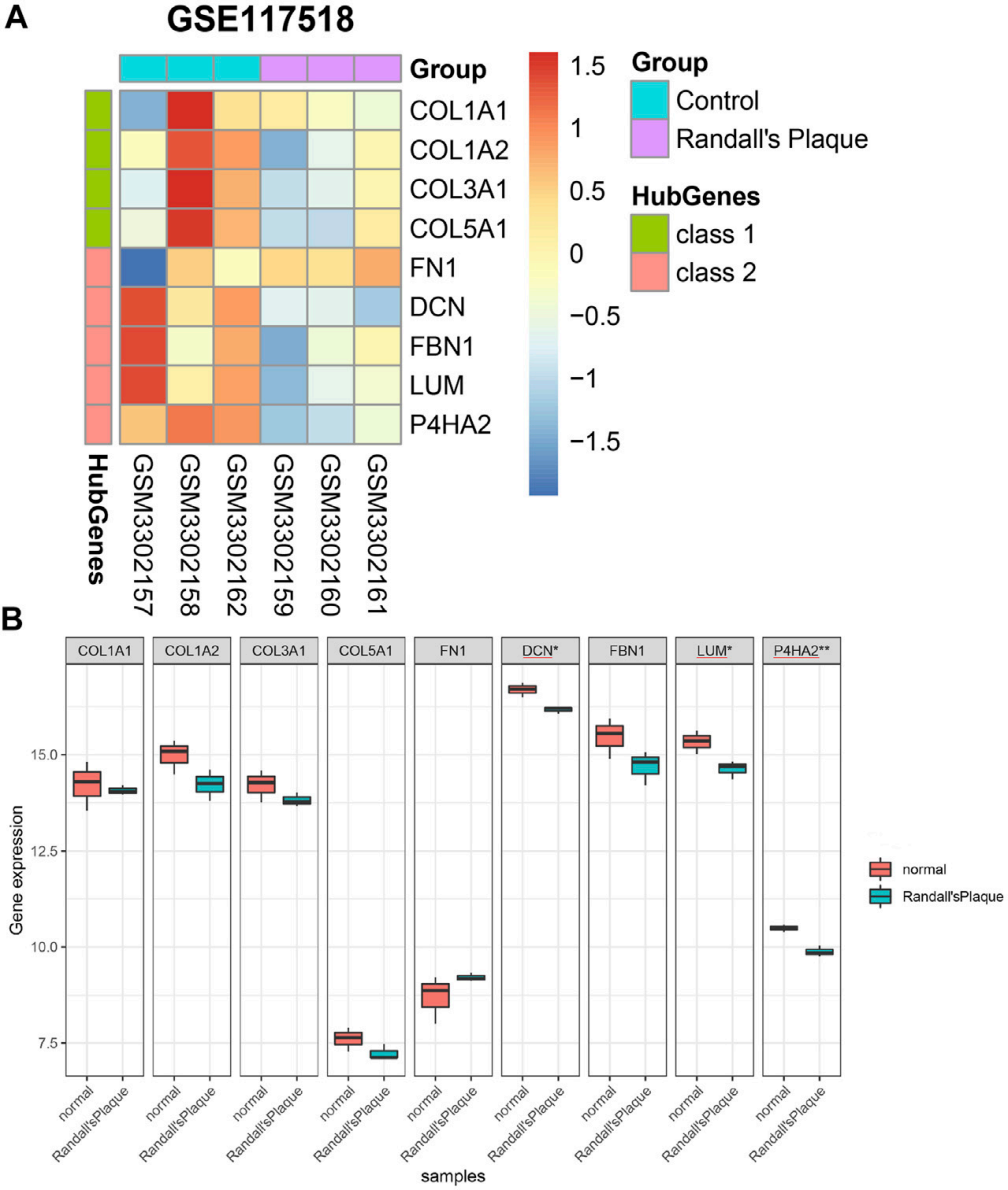
**(A)** Differential expression of nine hub genes in GSE117518. **(B)** Differential genes with significant differences between the normal and RP groups marked with asterisks.

Nine hub genes are confirmed to express in the GSE117518 gene matrix (Supplementary Table S3). They are illustrated in a heatmap (Figure 7A). It is noteworthy that the colors of DCN, FBN1, LUM, and P4HA2 are different in the control and RP groups. A minimum simple normalization Shapiro–Wilk test proves these nine hub genes of GSE117518 expression are normally distributed. Independent-sample *t*-test results found the difference is statistically significant in DCN, LUM, and P4HA2 (Figure 7B).

## 4 Discussion

Previous research into Randall’s plaque has investigated multiple fields, such as microscopic morphology (Evan et al., 2018) and metabolic disturbance (Coe et al., 2016). Recent experiments emphasized M1/M2 macrophage polarization and osteogenic reaction, which was associated with osteopontin (OPN), runt-related transcription factor 2 (Runx2), and bone osteocalcin (OCN) (Cui et al., 2020) in Randall’s plaque formation. Compared with former findings, our study concludes that DCN, LUM, and P4HA2 are downregulated in Randall’s plaque in coordination with the abnormal distribution of resting mast cells and M2 macrophages.

DCN, as the small leucine-rich proteoglycan (SLRP) family class I member, always serves as the ligand of receptor tyrosine kinases to restrict tumor proliferation (Santra et al., 2000) or modulate tumor angiogenesis signals (Järveläinen et al., 2015). Notably, SLRP class II member-LUM remains controversial in its anti-cancer effect and prognosis evaluation (Appunni et al., 2021). However, both DCN SLRP class II member-LUM display wound healing functions *via* angiogenesis, inflammation, and fibroblast activities (Karamanou et al., 2018; Pang et al., 2019). Unlike them, P4HA2 encodes a component of prolyl 4-hydroxylase (Gupta and Wish, 2017) as one member of the proline hydroxylases (PHDs) family, which catalyzes the formation of (2S,4R)-4-hydroxyproline in collagen (Gorres and Raines, 2010) and always mediates hypoxia-inducible factor-1 alpha (HIF-1α) degradation. As indicated by previous summaries, the formation of Randall’s plaque is associated with interstitial fibrosis (Evan et al., 2006; Marien and Miller, 2016). Additionally, Schaefer found that DCN exerts beneficial effects on tubulointerstitial fibrosis (Schaefer et al., 2002). In addition, as the substrate of hydroxylation modification by prolyl 4-hydroxylase, HIF-1α could promote tubular interstitial fibrosis (Kimura et al., 2008). Conversely, few experiments explored the LUM anti-fibrotic effect, although it is preferentially expressed in tubulointerstitium (Hsieh et al., 2014). These studies suggested that DCN and P4HA2 would ameliorate interstitial fibrosis and may play an important role in the progression of Randall’s plaque. Because BioGPS indicates these genes are highly expressed in extra-renal sites, we may ignore their functions in the kidney.

The formation of renal stones is divided into four stages: first, supersaturation-induced CaP crystal deposition; second, the growth and expansion of CaP permeate into the renal interstitium toward the papillary surface; third, cumulative crystal deposition destroys the papillary surface epithelium and directly protrudes into the urine in renal calyx; and finally, urinary CaOx gradually adheres, replaces the CaP, and becomes covered by urinary substance (Khan and Canales, 2015) (Supplementary Figure S1). Even though RP is thought to originate from the ascending thin limb at the Henle loop (Evan et al., 2018), the mechanism of its formation remains controversial. Currently, crystal deposition is considered to be similar to bone formation (Jia et al., 2014; Joshi et al., 2015). The proteomic analysis reveals that DCN and LUM are associated with the hydroxyapatite interaction proteins in bone (Zhou, 2007), and DCN could inhibit the hydroxyapatite-induced crystal growth (Sugars et al., 2003). Although the DCN level is low in the RP group, the relatively low level of DCN presumably weakens the competitive inhibit effect of other molecules, such as asporin, on hydroxyapatite mineralization (Kalamajski et al., 2009). Additionally, the deregulation of HIF-1α is associated with the activation of M2 macrophage polarization in CaOx nephrocalcinosis (Yang et al., 2020), which could be degraded *via* the proline hydroxylase P4HA2 family. Therefore, the decreasing P4HA2 level in the RP group may lift the restriction on HIF-1α expression.

In addition, renal stones are thought to be related to immune regulation. In 1999, de Water et al. noticed that renal or peripheral macrophages were recruited in kidney stone disease (de Water et al., 1999). In subsequent experiments, M2 macrophages, also called anti-inflammatory macrophages, were found to alleviate renal crystal deposition through CSF-1 stimulation (Taguchi et al., 2014), whereas M1 macrophages facilitated crystal development (Taguchi et al., 2016). Dominguez-Gutierrez et al. (Dominguez-Gutierrez et al., 2018) found that CaOx could induce monocytes into M1 macrophages in macrophage polarization. Indeed, the activation degree of macrophage phagocytosis ability varied among diverse substances like M-CSF or GM-CSF (Kusmartsev et al., 2016).

Despite widespread macrophage studies, other immune cells have not been investigated in Randall’s plaque in recent years. According to the correlation heatmap results, a comprehensive immune regulatory network illustrated underlying relationships among immune cells, limited by sample sizes. Only mast cells and M2 macrophages are pronounced in the violin plot. Indeed, the neutrophil–lymphocyte ratio has been proposed as a diagnostic biomarker in a kidney stone clinical survey (Mao et al., 2021). It is noteworthy that mast cells (MCs) are contradictory in animal kidney fibrosis model research (Madjene et al., 2015). Kim indirectly confirmed the MC protective effect in kidney fibrosis in that MC-deficient animals exhibited more severe tubular fibrosis than the MC-sufficient controls (Kim et al., 2009). These results contrast with the detrimental effect in mouse models (Summers et al., 2012). Therefore, in light of the potential relationship between Randall’s plaque and fibrosis, it is essential to extend the research into clinical studies and explore a therapeutic target for Randall’s plaque by elucidating the fibrosis mechanism with MC.

## 5 Conclusion

We assume *DCN*, *LUM*, and *P4HA2* may play a role in Randall’s plaque pathogenesis. Furthermore, M2 macrophages and resting mast cells are involved in the immune regulatory network in the formation of Randall’s plaque. These immune cell markers and hub genes could serve as potential biomarkers and provide new research directions.

## Data Availability

The original contributions presented in the study are included in the article/Supplementary Material; further inquiries can be directed to the corresponding author.

## Supplementary material

Supplementary Figure S1 Randall’s plaque formation in the renal papillae of kidney.
