# Supplementary figures and images for: Identification of the core genes in Randall’s plaque of kidney stone and immune infiltration with WGCNA network
Source: Front Genet. 2023 Feb 1;14:1048919. doi: 10.3389/fgene.2023.1048919 (PMC9931196; doi:10.3389/fgene.2023.1048919)

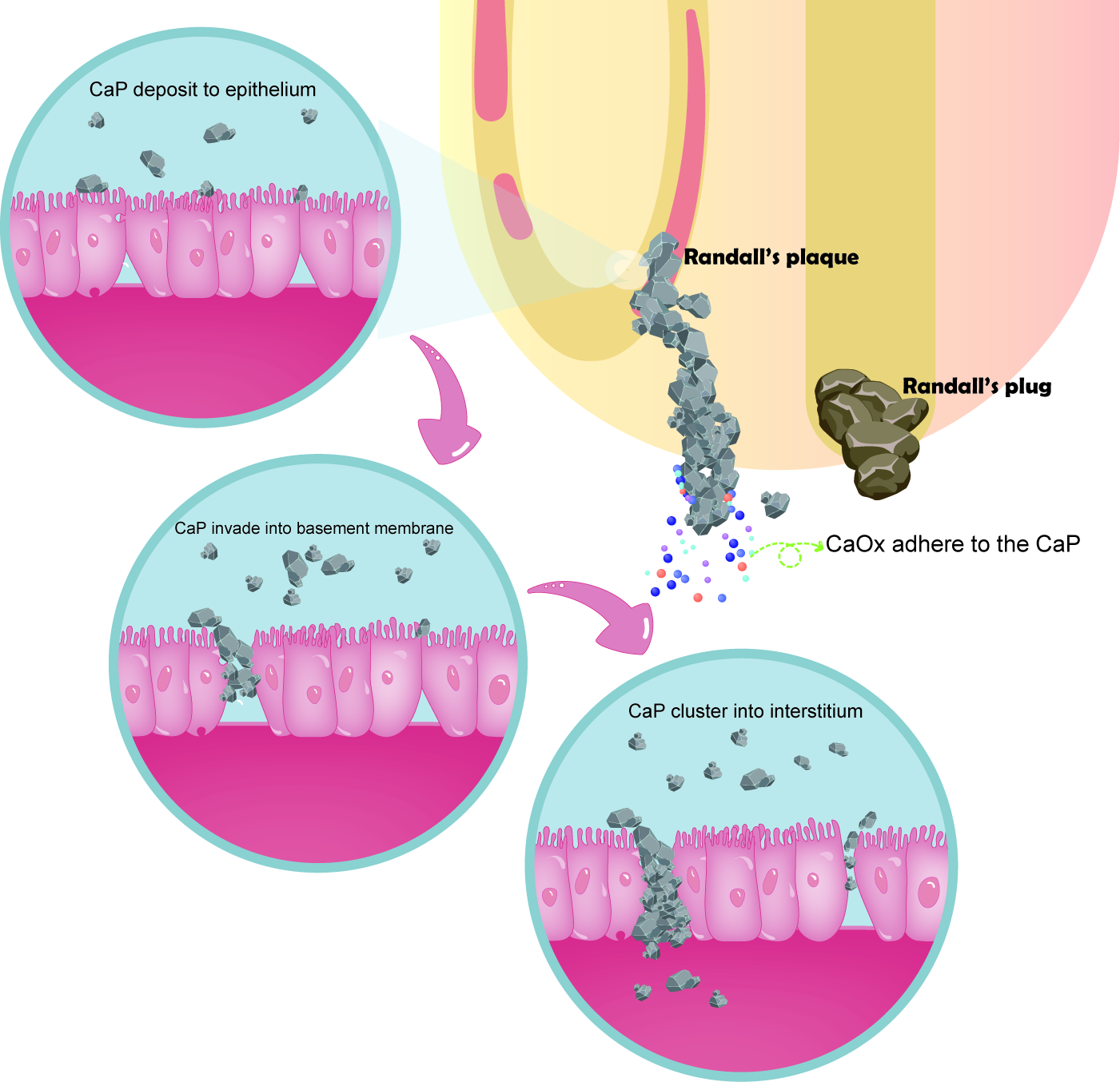

Supplement: Supplementary file 3 [file Image1.TIF]
